# Associations Between Weight-Related Measurements and Pubertal Markers in Norwegian Girls: The Bergen Growth Study 2

**DOI:** 10.1210/jendso/bvaf100

**Published:** 2025-06-04

**Authors:** Ingvild Særvold Bruserud, Mathieu Roelants, Ingvild Halsør Forthun, Kristin Viste, Robert Bjerknes, André Greger Madsen, Lawrence M Schell, Ninnie B Oehme, Petur B Juliusson

**Affiliations:** Children and Youth Clinic, Haukeland University Hospital, Bergen 5021, Norway; Faculty of Health and Social Sciences, Western University College of Applied Sciences, Bergen 5063, Norway; Department of Care, Flemish Government, 1000 Brussels, Belgium; Children and Youth Clinic, Haukeland University Hospital, Bergen 5021, Norway; Department of Clinical Science, University of Bergen, Bergen 5020, Norway; Department of Clinical Science, University of Bergen, Bergen 5020, Norway; Hormone Laboratory, Department of Medical Biochemistry and Pharmacology, Haukeland University Hospital, Bergen 5021, Norway; Children and Youth Clinic, Haukeland University Hospital, Bergen 5021, Norway; Department of Clinical Science, University of Bergen, Bergen 5020, Norway; Hormone Laboratory, Department of Medical Biochemistry and Pharmacology, Haukeland University Hospital, Bergen 5021, Norway; Department of Epidemiology and Biostatistics, University at Albany, Albany, NY 12222, USA; Children and Youth Clinic, Haukeland University Hospital, Bergen 5021, Norway; Children and Youth Clinic, Haukeland University Hospital, Bergen 5021, Norway; Department of Clinical Science, University of Bergen, Bergen 5020, Norway; Health Registry Research and Development, National Institute of Public Health, Bergen 5808, Norway

**Keywords:** puberty, development, menarche, maturation, pubertal assessment, pediatric endocrinology

## Abstract

**Context:**

Previous studies that have documented the association between puberty and overweight and obesity have typically used body mass index (BMI) as a measure of weight status, and menarche as outcome. Other markers of body weight or pubertal development are less studied.

**Objective:**

To characterize associations between weight-related anthropometric measurements and pubertal markers in Norwegian girls.

**Methods:**

A total of 646 schoolgirls aged 6 to 16 years from the Bergen Growth Study 2 were included. Pubertal status was assessed with ultrasound staging of breast development (USB), Tanner pubic hair stage, and menarche. Reproductive hormones were analyzed with liquid chromatography–tandem mass spectrometry and immunoassays. BMI, waist circumference, subscapular skinfolds (SSF), and percentage body fat measured by bioelectrical impedance (%BF) were converted to z-scores to study associations with pubertal status with logistic/linear regression adjusted for age (all pubertal markers) and Cox regression (menarche).

**Results:**

For all stages of breast development and all weight-related variables, girls with relatively low (< −1z) levels of anthropometric measurements were less likely (age-adjusted odds ratio [AOR] <1) and girls with high z-score levels were more likely (AOR >1) to have reached a particular stage compared to average-weight peers. Comparable associations were found for menarche. Pubarche was not significantly associated with weight-related measurements. Interestingly, serum concentrations of estrogens were lower in pubertal girls (USB2-5) with increasing SSF and %BF z-scores.

**Conclusion:**

Associations between all included weight-related anthropometric measurements and breast development and menarche, but not pubarche, were found strong and consistent. Our findings indicate complex associations between reproductive hormones and weight status.

During the last decades, there have been global trends of both earlier onset of female puberty as well as increased prevalence of overweight and obesity in the pediatric population [1]. Both early onset of puberty and pediatric obesity are associated with adverse health outcomes [2, 3]. Several studies have shown that high body mass index (BMI) for age is a strong risk factor for attaining pubertal markers such as breast development or menarche at a younger age [4-6]. However, increased BMI may explain earlier puberty onset only in part, since the trend toward earlier maturation seems to affect girls irrespective of their BMI [7, 8].

BMI is the most commonly used indicator of weight status in children [9], but it has limitations in defining adiposity [10, 11]. The BMI cannot discriminate between fat-free (lean) mass and body fat, and the body composition may vary considerably in children with a similar BMI [12]. Weight-related anthropometric measurements such as waist circumference (WC) and skinfolds are measures that add complementing information to describe body composition. Waist circumference correlates with truncal adiposity [13], and the subscapular skinfolds (SSF) correlate with total body fat [10]. Although it has been recognized that these measurements are correlated [14], less knowledge exists on how they are associated with pubertal development in a healthy (ie, nonclinical) population.

The current study is a part of the Bergen Growth Study 2, a cross-sectional study conducted in 2016 that documented pubertal development in Norwegian children. The study used ultrasound to assess breast maturation in girls in addition to traditional Tanner stages, and data on weight-related anthropometric measures and blood samples for measurements of pubertal hormones were collected [15].

The aim of the present study was to investigate the association and impact of relatively low and high z-scores levels of different anthropometric weight-related measurements, namely BMI, WC, SSF, and percentage of body fat (%BF) estimated with bioelectrical impedance analysis (BIA), with breast and pubic hair development, and menarche, in a representative sample of healthy Norwegian girls. We also aimed to characterize and study the associations of the serum concentrations of reproductive hormones across categories of weight status in pre-pubertal and pubertal girls. We hypothesized that the weight-related measures would associate differently with the markers of puberty.

## Methods

### Study Subjects

The Bergen Growth Study 2, a cross-sectional study, recruited 678 girls aged 6 to 16 years from public schools in Bergen, Norway, in 2016 (for details [8, 15]). Girls with chronic diseases or conditions likely to affect growth or development (n = 27) were excluded. The final sample used for analyses includes 646 girls, due to missing clinical data in 4 girls. The mean (range) age was 11.1 (6.1-16.2) years. A parental questionnaire was obtained from 482 girls (74%) and included questions regarding chronic illnesses and demographic data such as ethnic origin. Of these, there were 374 (81%) sets of parents where both were born in Norway.

### Pubertal Assessments

In the Bergen Growth Study 2, ultrasound was used to describe the development of glandular breast tissue throughout puberty, and to classify development based on characteristic morphological changes [16, 17]. Breast development was evaluated by a female nurse (I.S.B.) using ultrasound breast staging (USB), where stages 0 and 1 represent immature, pre-pubertal stages, stages 2 to 4 indicate pubertal development, and stage 5 is considered mature [16]. The girls were examined in the supine position and all ultrasound images were captured in a standardized mid-sagittal plane.

Furthermore, breast and pubic hair development were assessed using 5 Tanner stages as described by Marshall and Tanner [18]. Stage 1 represents the earliest stage indicating absence of breast and pubic hair development, while the subsequent stages indicate the onset (thelarche or pubarche, stage 2) and successive pubertal development. The clinical breast assessments included both visual inspection and palpation. Data on menarche were recorded by asking the participants if they had experienced their first menstruation (and if yes, to recall the specific month and year).

### Anthropometric Measurements

Height was measured according to standardized procedures with a Harpenden Portable Stadiometer (Holtain Ltd Crosswell, UK) and recorded to the nearest 0.1 cm [19]. Weight was measured in light clothing on a combined electronic scale and bioelectrical impedance analysis machine (BIA) (Tanita MC-780MA, Tanita Corp. of America, Inc. Illinois, USA) and recorded with a precision of 0.1 kg. The same machine estimated the percentage of body fat (%BF). BMI was calculated by dividing weight (in kg) by the square of height (in meters).

SSF was measured on the left-hand side of the back with a Holtain Tanner/Whitehouse Skinfold Caliper, (Corswell, UK) [20]. The waist circumference (WC) was measured with a nonelastic metallic tape in a horizontal position half-way between the upper part of the hip (iliac crest) and the lowest rib. Z-scores, equivalent to SD scores, for BMI, SSF, and WC were calculated using sex and age specific national growth references [21-23]. The z-scores for %BF were calculated based on the UK references [24].

### Laboratory Analyses

Venous blood samples were collected between 8 Am and 2 Pm (average time 10:57 Am). The samples were analyzed at the Hormone Laboratory at the Department of Medical Biochemistry and Pharmacology, Haukeland University Hospital, Bergen, Norway. Serum testosterone, estradiol (E2) and estrone (E1) were analyzed by liquid chromatography–tandem mass spectrometry (LC-MS/MS) as described previously [25-27]. The analytical coefficient of variation (CV) for E1 and E2 was 9.1% (range, 1.7-153.3 pmol/L), and the lower limit of quantification (LLOQ) was 0.58. For testosterone, the CV was 4% with a LLOQ of 0.02 nmol/L. Basal levels of follicle-stimulating hormone (FSH) (CV 5% at 17 IU/L), luteinizing hormone (LH) (CV 7% at 10 IU/L) and sex-hormone binding globulin (SHBG) (CV 6% at 60 nmol/L) were analyzed by chemiluminescence using Siemens IMMULITE 2000 XPi. To quantify serum leptin, enzyme-linked immunosorbent assay kits were used (Mediagnost Cat# E07, RRID: AB_2813737). The inter-assay CV was 5% at 8.3 µg/L for leptin (LLOQ 1-100 µg/L). The LLOQ was 0.1 IU/L for FSH and LH and 2 nmol/L for SHBG. Serum concentrations below the LLOQ (for LH and FSH) were replaced with 0.1 in the analysis. To investigate the hormonal levels between girls in sensitivity analyses, z- scores for the serum concentrations of E1, E2, LH, FSH, LH/FSH ratio, leptin, testosterone, and SHBG were calculated using previously published data [28]. Concentration of free E1 and free E2 were calculated based on the formula by Mazer [29] and free testosterone was calculated based on the formula by Vermeulen [30].

### Statistical Analyses

All anthropometric variables (BMI, WC, SSF, %BF) were categorized as low (< −1 z-score), average (−1 ≤ z ≤ 1), or high (>1 z) and used as predictor variables. Binary logistic regression with age as covariate was used to study the likelihood of being in a pubertal level of breast (B ≥ 2) or pubic hair development (PH ≥ 2), or for being in stages B3, B4, B5, or PH3, PH4, PH5 in girls with low or high levels of anthropometric measurements compared to girls with average levels. When investigating the onset of breast development (B ≥ 2), girls between 8 and 13 years were included, while girls between 8 and 14 years were included for the analyses for onset of pubic hair (PH ≥ 2). This was done to exclude the ages where all girls were pre-pubertal, or all girls were *in* puberty. The mean (SD) ages at pubertal onset in the subgroups of girls with low, average, and high anthropometric measures were estimated with probit regression.

In addition to the separate analysis of BMI, WC, SSF, and %BF we used multiple logistic regression to investigate the effect of all weight-related markers combined, with age as covariate on the onset of puberty in a fully adjusted model.

The association between weight-related anthropometric measures (BMI, WC, SSF, %BF) and the timing of menarche was analyzed with Cox regression using the recalled age at menarche when applicable and censoring at the age at examination when not. The anthropometric variables were categorized as described above. Results are presented as hazard ratios (from the Cox proportional hazards model) in an unadjusted model for BMI, WC, SSF, and %BF z-scores, and in a fully adjusted model including all 4 variables. Kaplan-Meier analyses were used to estimate median (95% CI) age at menarche in the subgroups of girls with low, average, or high levels of the anthropometric measures. Kaplan-Meier plots were used to illustrate these associations. For the analyses of the timing of menarche, only girls above 11 years were included to exclude the ages where no girls were expected to have menarche.

The median and range of the hormonal serum concentrations were calculated and reported in girls with low, average, and high BMI, WC, SSF and %BF z-scores for descriptive purposes. Linear regression was used to investigate the concentration of reproductive hormones across different levels of anthropometric measurements. For these analyses, the hormone serum concentrations were log-transformed and used as dependent variables, age and grouped anthropometric measurements were independent variables. The analyses were conducted separately for girls with a pre-pubertal stage (USB < 2) and in pubertal stages (USB ≥ 2). In pubertal girls, ultrasound stage was included as a covariate. Data on the menstrual cycle among menstruating girls was not recorded, which limits our analysis of hormone serum concentrations to pre-menarcheal girls. In a sensitivity analysis, however, girls with menarche were included and menarche (no/yes) used as independent variable together with USB stage. Statistical analyses were performed in IBM SPSS statistics version 25 and R version 4.3.2 (R foundation for statistical Computing).

### Ethical Considerations

The Bergen Growth Study 2 was approved by the Norwegian Ethical Committee for Medical and Health Research Ethics West (REC-WEST 2015/128). Before inclusion, written consent form was obtained from a (responsible) caregiver for each participant and from adolescents older than 12 years. The included children provided verbal consent before examinations.

## Results

Of 678 girls, 364 (57%) girls had started pubertal breast development (USB ≥ 2). A total of 180 (48%) girls (of 372 with Tanner PH assessment) had started pubic hair development, and a total of 193 (30%) (of 643) girls had experienced menarche. The distribution of girls with a relatively low (< −1 z), average (−1 ≤ z ≤ 1) or high (>1 z) measurement of BMI, WC, SSF, and %BF are shown in Table 1. Based on the classification by the International Obesity Task Force [9, 31], a total of 47 (7.3%) girls were underweight, 503 (78.1%) had an average weight, 80 had overweight (12.4%) and 14 (2.2%) had obesity. Serum levels were available from 572 (E1), 557(E2), 612 (LH), 611(FSH), 613 (SHBG), and 613 (testosterone) girls.

**Table 1.** Age at USB2 and Tanner PH2 in girls with a low, average, or high body mass index, waist circumference, subscapular skinfold, and body fat percent z-scores (probit analysis), and logistic regression of reaching pubertal status in these groups, adjusted for age

			Thelarche USB ≥ 2 (≥8 years and ≤13 years)						Pubarche Tanner ≥ PH2 (≥8 years and ≤14 years)			
		n	Mean (SD) age	AOR	(95% CI)	*P* value		n	Mean (SD) age	AOR	(95% CI)	*P* value
BMI z-scores	Low	55	11.1 (1.1)	0.29	(0.12, 0.65)	.003	Low	32	11.6 (1.5)	0.32	(0.09, 1.01)	.060
	Average	249	10.3 (1.2)				Average	157	10.8 (1.2)			
	High	52	9.3 (1.1)	3.68	(1.59, 9.02)	.003	High	46	10.8 (0.8)	0.95	(0.36, 2.46)	.915
WC z-scores	Low	38	11.0 (1.3)	0.38	(0.14, 0.96)	.046	Low	27	11.4 (1.1)	0.41	(0.11, 1.42)	.170
	Average	272	10.3 (1.2)				Average	172	10.9 (1.3)			
	High	46	9.1 (1.1)	6.03	(2.30, 17.97)	.001	High	36	10.7 (0.9)	1.31	(0.47, 3.70)	.606
SSF z-scores	Low	59	10.9 (1.1)	0.36	(0.15, 0.81)	.016	Low	38	11.3 (1.4)	0.46	(0.14, 1.44)	.193
	Average	219	10.3 (1.3)				Average	136	10.8 (1.2)			
	High	79	9.6 (1.1)	2.24	(1.13, 4.52)	.022	High	60	10.8 (1.1)	0.98	(0.40, 2.37)	.963
%BF z-scores	Low	47	10.4 (0.9)	0.84	(0.37, 1.88)	.667	Low	32	10.8 (0.7)	1.03	(0.33, 3.11)	.965
	Average	267	10.3 (1.4)				Average	180	10.9 (1.3)			
	High	41	9.4 (1.2)	3.57	(1.48, 9.12)	.006	High	23	10.8 (0.9)	1.24	(0.35, 4.53)	.741

Age data are mean (SD), odds ratios are adjusted for age (AOR). Anthropometric measurements grouped as low (< −1 z-score), average (−1 ≤ z-score ≤ 1) or high (>1 z-score).

Abbreviations: %BF, body fat percent; AOR, age-adjusted odds ratio; BMI, body mass index; SSF, subscapular skinfold; Tanner PH, Tanner stages of pubic hair; USB, ultrasound determined breast stage; WC, waist circumference.

### Breast Development

Girls with a low level of BMI, WC, and SSF z-scores had a statistically significant lower age-adjusted odds ratio (AOR) of having reached thelarche (USB ≥ 2) compared to girls with average level z-scores (Table 1). Girls with a high level of BMI, WC, SSF, and %BF z-scores had a statistically significant higher AOR of having reached thelarche (AOR > 1). In the fully adjusted models with all 4 anthropometric measurements, only a low BMI z-score (AOR: 0.35 [95% CI 0.13, 0.94], *P* = 0.041) was significantly associated with a lower probability of having reached stage USB ≥ 2.

For the more advanced stages of breast development (USB3, USB4, and USB5), both a low and high BMI were also significantly associated with lower (AOR < 1) and higher (AOR > 1) odds of being in each stage, respectively (Supplementary Fig. S1 [32]). Similar trends were seen for WC and SSF z-scores. Results for breast development according to the Tanner B stage are shown in Supplementary Table S1 and Supplementary Fig. S2 [32]. No association between anthropometric markers and Tanner B ≥ 2 was statistically significant in a fully adjusted model despite consistent patterns of an AOR < 1 in girls with a low anthropometric status and AORs > 1 in girls with a high anthropometric status (data not shown).

### Pubic Hair

The impact of low or high anthropometric measurements on pubarche (Tanner PH ≥ 2) was also not statistically significant, despite a generally consistent lower AOR in girls with a low BMI, WC, SSF, or %BF z-scores and higher AOR in girls with high z-score (Supplementary Fig. S3 [32]). In a fully adjusted model, no significant association was observed for pubarche (Tanner PH ≥ 2) (data not shown).

### Menarche

**Figure 1.**
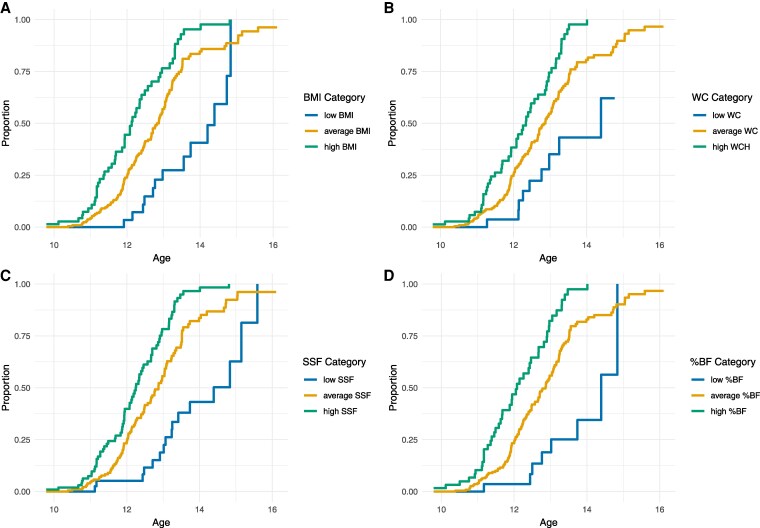
Kaplan-Meier curves for age at menarche in healthy girls studied the in Bergen Growth Study 2. The curves show that menarche occurs later in girls with a relatively low (< −1z-score) level, and at later age in girls with high (>1 z-score) level of body mass index (BMI) (panel A), waist circumference (WC) (B), subscapular skinfolds (SSF) (C), and body fat percent (%BF) (D).

The median age (95% CI) at menarche estimated with Kaplan-Meier analysis was 12.8 (12.6, 13.0) years for all girls. Cox regression analyses showed that low and high values of BMI, WC, SSF, and BF% z-scores were associated with a lower and higher likelihood of having menarche, respectively (Table 2). When adjusting for all other covariates, only a low SSF and high %BF z-score were significantly associated with the timing of menarche compared with average-weight girls (Table 2 and Fig. 1).

**Table 2.** Kaplan-Meier estimates of age, unadjusted and fully adjusted hazard ratios (HR) from Cox regression, at having menarche according to z-scores for BMI, WC, SSF, and %BF for 297 girls in Bergen Growth Study 2 in 2016

				Unadjusted			Fully adjusted		
	N	Mean (SD) age	Median	HR	(95% CI)	*P*	HR	(95% CI)	*P*
**BMI z-score**									
Low	36	14.1 (0.8)	14.4	0.37	(0.21, 0.65)	<.**001**	0.62	(0.33, 1.19)	.149
Average	202	12.8 (1.4)	12.9						
High	60	12.0 (0.6)	12.1	1.98	(1.42, 2.77)	<.**001**	1.10	(0.63, 1.90)	.747
**WC z- score**									
Low	27	14.1 (1.1)	14.4	0.41	(0.21, 0.81)	.**011**	0.99	(0.45, 1.90)	.982
Average	209	12.9 (1.4)	12.9						
High	62	12.0 (0.6)	12.3	2.05	(1.46, 2.87)	**<**.**001**	0.89	(0.50, 1.57)	.677
**SSF z-score**									
Low	39	14.2 (1.4)	14.39	0.36	(0.21, 0.61)	**<**.**001**	0.48	(0.28, 0.85)	.**012**
Average	169	12.9 (1.3)	12.87						
High	89	12.1 (0.5)	12.25	2.04	(1.5, 2.8)	**<**.**001**	1.49	(0.90, 2.45)	.118
**%BF z-score**									
Low	29	14.5 (2.3)	14.4	0.28	(0.14, 0.57)	**<**.**001**	0.55	(0.24, 1.29)	.170
Average	213	12.8 (1.4)	12.9						
High	55	11.9 (0.01)	12.1	2.47	(1.73, 3.52)	**<**.**001**	1.69	(1.03, 2.78)	.**039**

Age data are mean (SD). Girls ≥11 years are included in analyses. Probit regression for mean (SD) age at having menarche. Kaplan-Meier estimates of median ages at menarche in groups. Probit mean age (SD) at menarche for all girls is 12.8 (1.35) years.

Anthropometric measurements grouped as low (< −1 z), average (−1 ≤ z ≤ 1) or high (>1 z).

BMI, WC, SSF and %BF and age were included in the fully adjusted model.

Values in bold indicate that analysis reached statistical significance.

Abbreviations: %BF, percentage body fat; BMI, body mass index; HR, hazard ratio; SSF, subscapular skinfold; WC, waist circumference.

### Serum Hormone Concentrations

**Figure 2.**
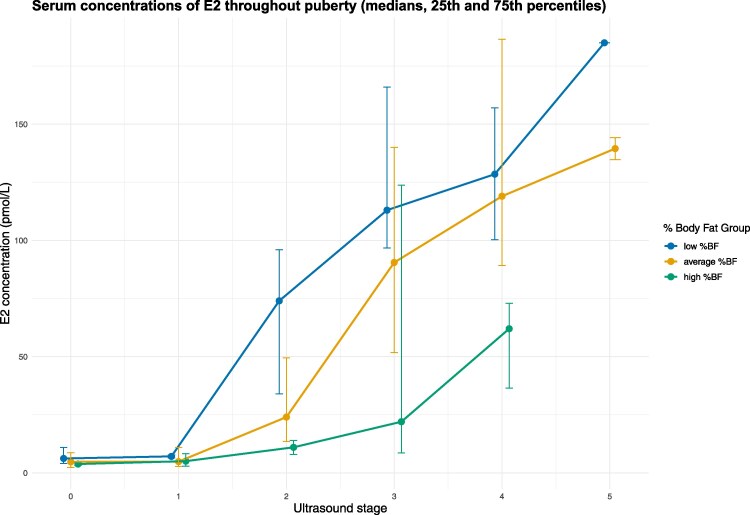
Plot showing median (error bars represent 25th and 75th percentiles) estradiol (E2) concentrations (pmol/L) in pre-menarcheal girls according to ultrasound breast stages 0 to 5 for 395 healthy girls aged 6 to 16 years included in the in Bergen Growth Study 2 in 2016.

Before pubertal onset, the serum concentrations of E1, E2, FSH, and LH increased with age (Supplementary Tables 2-4 [32]) but were not significantly associated with the anthropometric variables. In girls who started breast development but did not yet reach menarche, serum concentrations of these hormones were higher in those who had a low anthropometric z-score, and lower in girls with a high anthropometric z-score. Linear regression analysis, adjusted for age and stage of breast development (USB), showed that girls with increasing z-score for any anthropometric measurement tended to have lower levels of reproductive hormones. Significant associations were found for girls with increasing levels of SSF and %BF z-scores and lower serum levels of E1 and E2. For %BF z-scores, significant associations were also found for free E2, FSH, and LH (regression coefficients are shown in Table 3; illustration of E2 according to USB stage in Fig. 2). Serum concentrations of SHBG and leptin were significantly associated with the level of all anthropometric measurements both before and after pubertal onset

**Table 3.** Associations between BMI, WC, SSF, or %BF and hormonal serum concentrations adjusted for age and pubertal stage in 170 pubertal girls (ultrasound breast stage 2-5) before menarche in the Bergen Growth Study 2

	BMI	WC	SSF	%BF
Hormone	Regression coefficient (95% CI)	Regression coefficient (95% CI)	Regression coefficient (95% CI)	Regression coefficient (95% CI)
Estrone E1 (pmol/L)	0.88 (0.74, 1.04)	0.88 (0.74, 1.04)	0.83 (0.72, 0.96)*^c^*	0.74 (0.63, 0.87)*^b^*
Estradiol E2 (pmol/L)	0.77 (0.58, 1.02)	0.75 (0.55, 1.01)	0.72 (0.56, 0.93)*^c^*	0.56 (0.43, 0.73)*^b^*
Free estradiol*^d^* (pmol/L)	0.87 (0.65, 1.15)	0.88 (0.65, 1.18)	0.84 (0.65, 1.08)	0.61 (0.47, 0.8)*^b^*
FSH (IU/L)	0.92 (0.79, 1.07)	0.88 (0.75, 1.04)	0.93 (0.81, 1.06)	0.75 (0.65, 0.87)*^b^*
LH (IU/L)	0.99 (0.71, 1.38)	0.84 (0.60, 1.19)	0.78 (0.58, 1.04)	0.56 (0.41, 0.76)*^b^*
Testosterone (nmol/L)	0.996 (0.86, 1.15)	0.99 (0.85, 1.14)	0.92 (0.81, 1.05)	0.88 (0.77, 1.02)
Free testosterone*^e^* (nmol/L)	1.18 (1.00, 1.39)*^c^*	1.23 (1.04, 1.46)*^c^*	1.12 (0.97, 1.30)	1.02 (0.86, 1.20)
SHBG (nmol/L)	0.79 (0.70, 0.89)*^b^*	0.74 (0.65, 0.83)*^b^*	0.76 (0.69, 0.84)*^b^*	0.82 (0.73, 0.92)*^b^*
Leptin (µg/L)	1.83 (1.54, 2.17)*^a^*	2.13 (1.81, 2.52)*^a^*	2.03 (1.78, 2.33)*^a^*	2.02 (1.72, 2.37)*^a^*

Anthropometric measurements were grouped as relatively low (<−1z), average (−1 ≤ z ≤ 1) or high (>1 z) levels.

Abbreviations: %BF, percentage body fat; BMI, Body mass index; SSF, subscapular skinfold; WC, waist circumference. Regression coefficients and *P*-values derive from linear regression with anthropometric variable (low (< −1 z- score). average (−1 ≤ z-score ≤ 1) or high (>1 z-score) level), ultrasound breast stage (USB2-USB5) and age as covariates, and log-transformed hormone serum concentrations as dependent variables. Only girls without menarche are included.

^a^ *P* value < .001.

^b^ *P* value < .005.

^c^ *P* value < .05.

^d^Free estradiol is calculated based on the formula by Mazer [29].

^e^Free testosterone was calculated based on the formula by Vermeulen et al [30].

## Discussion

In the present study we found strong and consistent associations between all the included weight-related anthropometric markers and the timing of pubertal development assessed with ultrasound to determine glandular breast development, Tanner stages, and serum hormone concentrations. These associations were upheld throughout the more advanced breast maturational stages. No statistically significant relationship was found between anthropometric measurements and pubarche. Interestingly, we report a negative association between anthropometric measurements, in particular skinfolds and %bodyfat, and hormone serum concentrations in pre-menarcheal pubertal girls.

The associations between BMI, WC, SSF, and %BF z-scores and thelarche showed quite similar associations across weight-related measurements and weight groups in all stages of breast development, although not always statistically significant. We did not observe stronger relationships between more direct measurements of fat mass, such as SSF and %BF, and breast development compared to BMI and WC and puberty. These findings are consistent with previous research exploring associations between BMI and timing of pubertal development in girls [4, 5, 33-35].

Pubarche, an indicator of androgen activation, was not significantly associated with weight-related anthropometric traits, despite comparable trends across high and low measurements as observed for thelarche and menarche. Both cross-sectional [34, 36] and longitudinal [37] studies have noted associations between BMI and earlier pubarche, but findings are inconsistent. A large longitudinal US study with over 120 000 children, found that girls with obesity at age 5 to 6 years had a higher risk of both having earlier breast and pubic hair development [37]. Conversely, Denzer et al (2007) reported a normal adrenarche timing in girls with obesity (n = 650) in a cross-sectional study [38]. Another longitudinal study observed that the steepest increase in BMI, skinfolds, and percent body fat occurred after thelarche (n = 1166) [39]. These findings, along with ours, indicate that the increase in measurements of body composition may be more closely associated to the hypothalamic-pituitary-gonadal (HPG) axis than the hypothalamic-pituitary-adrenal axis. A possible role for adipose tissue aromatase has also been suggested in these processes, but this hypothesis is not supported by a recent longitudinal study investigating the impact of body fat on breast development in obese girls [40].

Interestingly, our results showed an inverse association between SSF and %BF z-scores and E1 and E2 in pre-menarcheal pubertal girls. The %BF was also negatively associated with gonadotrophin levels. Several studies have published similar findings, reporting that pubertal girls with higher BMI or %BF have with lower levels of estradiol or LH [35, 41, 42], and other studies have reported a reduced nocturnal rise in LH in early pubertal girls with obesity compared with girls with average weight when adjusted for Tanner B stage [43, 44]. In a recent study, however, no associations between total body fat percentage and estrogen level in pubertal girls were found [45]. It has previously been discussed that aromatase in adipose tissue may be associated with the timing of puberty in girls [46, 47]. This could potentially explain, partly, earlier maturation despite similar or lower levels of estradiol or LH in girls with overweight and obesity [42, 48]. However, our findings from pubertal girls do not support fat aromatization as a relevant mechanism, as the estrogen concentrations were lower in girls with high SSF and %BF z-scores.

Based on our cross-sectional data, we can only speculate on the mechanisms behind our findings. Higher levels of adiposity may impact the endocrine functions by altering the activity of the HPG axis, as previously discussed [35, 41]. Adiposity might impact the onset and the progression of puberty in distinct ways. It may contribute to earlier onset of mammary gland maturation (thelarche), perhaps by requiring less stimulus from the HPG axis, and at the same time associates with lower estrogen levels in later stages of pubertal development. In our study, the serum concentrations of E2 among the pubertal girls with high levels of %BF and SSF were low. We cannot rule out the possibility that some girls may have experienced transient thelarche in our study, a breast maturation without a central activation of the HPG axis. However, current literature does not support that adiposity mediate transient thelarche [49, 50]. Therefore, while this explanation seems unconvincing, we speculate that girls with varying levels of body fat may experience differences in estrogen sensitivity. This may align with Bordini et al suggesting a weaker hypothalamic drive to puberty in girls with excess weights [43]. And finally, although it is generally accepted that there is a link between estrogens and adiposity and fat distribution, the exact mechanisms are not fully understood [51]. There is a need for more research on the endocrine basis of how obesity modulates pubertal development.

### Strengths and Limitations

We applied ultrasound to determine the pubertal breast development, in addition to Tanner B staging in our study, possibly reducing the risk of misinterpreting adipose tissue as glandular tissue of the breast. In this study we used LC-MS/MS assays to measure all steroid hormones. This methodology is recommended due to its high selectivity and reduced risk of cross reactivity with other steroid hormone metabolites. High sensitivity was an advantage when investigating the pre-pubertal hormone concentrations, in particular. We included several weight-related measures (WC, SSF, and %BF) to provide a more comprehensive assessment of body composition. While dual energy x-ray absorptiometry (DXA) is considered the “gold standard” and preferred method when assessing body composition [52], its use is hampered by availability when conducting larger studies. Body fat % measured with BIA is, in general, considered to be an acceptable alternative, also in children [53, 54]. Future studies may incorporate standardized measurement protocols, including, for example, hydration when using BIA to estimate body fat distribution in children. The reference to determine z-scores for body fat %, was the study by McCarthy et al [24] and may explain some variance in our results. The age span of our study population was appropriate to study the onset of puberty and covered a range of girls at different pubertal stages. The included girls had predominantly healthy weight, and only 2.2% had obesity, highlighting thus associations between pubertal development and normal variations in body weight rather than effect of pathological weight gain or adiposity. In sensitivity analyses based on a subset of girls for whom a questionnaire was available, we adjusted for mothers’ age at menarche, socioeconomic status (with highest educational level of both parents as indicator), and ethnicity. There was a statistically significant effect of mothers age at menarche and origin, but inclusion had only minor impact on the reported effect sizes of BMI z-score on pubertal onset, and did not alter our conclusions (not shown).

The current study is limited by the cross-sectional design that can only show associations, and not prove causation, for example, girls may have changed their weight status between the time of examination and the timing for entering the stages. Including older girls (>16.5 years) would nevertheless have improved the study's ability to investigate the ages when all girls had reached mature stages of breast development. Another consideration is the varying hour of the day the blood samples were collected (8 hours to 14 hours), which may be considered in clinical practice. Data about the girls' menstrual cycles were not available among menstruating girls, limiting our analysis of hormone serum concentrations to pre-menarcheal girls. Having serum values of DHEA-S could have informed further about the pubertal maturation before thelarche [42] but were not available.

## Conclusions

All weight-related measurements showed significant associations to pubertal development, but BMI exhibited the most robust association to pubertal breast development. Although higher anthropometric levels were clearly associated with increased risk of having advanced in pubertal development, our findings also indicated an inverse association between serum concentrations of sex hormones and SSF and %BF z- scores. By decomposing explanatory variables for the timing and progression of pubertal development, namely anthropometric variables, and hormonal serum concentrations in relation to breast development, menarche and pubic hair development, we find that high levels of anthropometric measurements are unlikely to accelerate pubertal maturation through increased hormone levels in our cohort.

## Data Availability

Restrictions apply to the availability of data generated or analyzed during this study to preserve patient confidentiality or because they were used under license. The corresponding author will on request detail the restrictions and any conditions under which access to some data may be provided.

## Abbreviations

%BF: percentage of body fat
AOR: age-adjusted odds ratio
BIA: bioelectrical impedance analysis
BMI: body mass index
CV: coefficient of variation
E1: estrone
E2: estradiol
FSH: follicle-stimulating hormone
HPG: hypothalamic-pituitary-gonadal
LC-MS/MS: liquid chromatography–tandem mass spectrometry
LH: luteinizing hormone
LLOQ: lower limit of quantification
PH: pubic hair stage
SHBG: sex-hormone binding globulin
SSF: subscapular skinfold
USB: ultrasound breast stage
WC: waist circumference
